# Meta-analysis of the effect of racial discrimination on suicidality

**DOI:** 10.1016/j.ssmph.2022.101283

**Published:** 2022-11-03

**Authors:** Bruno Messina Coimbra, Chris Maria Hoeboer, Jutka Yik, Andrea Feijo Mello, Marcelo Feijo Mello, Miranda Olff

**Affiliations:** a Amsterdam UMC, University of Amsterdam, Department of Psychiatry, Amsterdam Public Health Research Institute and Amsterdam Neuroscience Research Institute, Amsterdam, the Netherlands; b Program for Research and Care on Violence and PTSD (PROVE), Department of Psychiatry, Universidade Federal de São Paulo (UNIFESP), São Paulo, Brazil; c ARQ National Psychotrauma Centre, Diemen, the Netherlands

**Keywords:** Racial discrimination, Racism, Mental health, Ethnicity, Minority groups, Public health, Suicide

## Abstract

Racial discrimination (RD) is unfair treatment of individuals based on race or ethnicity. It is a pervasive and increasing phenomenon in the lives of many individuals with deleterious effects on mental health. Research implicates RD in diminished well-being, lower life satisfaction and self-esteem, and mental health disorders. Furthermore, there have been reports that minorities and marginalized groups exposed to RD are at a higher risk of suicide. Given that RD negatively impacts mental health and that suicide is a major public health concern, we meta-analytically reviewed the literature to investigate whether RD is associated with suicidal ideation (SI) and suicide attempt (SA). We identified 43 eligible articles investigating the association between RD and suicidality through PubMed, Embase, PsycINFO and Scopus, from which we pooled 39 effect sizes for SI (58,629 individuals) and 15 for SA (30,088 individuals). Results demonstrated that RD has a small but significant effect both on SI (*r* = 0.16, 95% CI: 0.12 to 0.19; *p* < 0.0001) and on SA (*r* = 0.13, 95% CI: 0.02 to 0.23; *p* = 0.018). We found no indication of publication bias, and fail-safe tests confirmed the robustness of the results. Furthermore, we tested the moderating effects of several study characteristics (e.g., age, race, RD and SI time frame assessment, and categorization of RD measures). The only study characteristic to moderate the effect of RD on SI was SI time frame assessment (*r* = 0.07; 95% CI: 0.015 to 0.12; *p* = 0.01). Our findings suggest that SI and SA are phenomena that may be influenced by exposure to RD. Thus, individuals that are discriminated based on race may develop more suicidal thoughts and an increased likelihood of attempting suicide. These findings underscore the need for more prevention and intervention efforts to attenuate the effect of RD on suicidality.

## Highlights

• We conducted a meta-analysis of 62,349 individuals.
• We pooled effect sizes from 43 studies from seven countries.
• Racial discrimination has a statistically significant effect on suicidal ideation (*r* = 0.16, *p* < 0.0001).
• Racial discrimination has a statistically significant effect on suicide attempt (*r* = 0.13, *p* = 0.018).

## 1 Introduction

Racial discrimination (RD) is unfair treatment of individuals or groups based on race and related classifiers such as ethnicity and nationality (Pager & Shepherd, 2008; Paradies et al., 2015). It is a widespread, pervasive phenomenon, strongly intertwined in societal interrelations and an inherited aspect in the daily lives of many individuals (Bourabain & Verhaeghe, 2021). Evidence suggests that RD is a system embedded in social dynamics, resulting in heightened hostility toward minority and disadvantaged groups in society and the stratification of racial and ethnical groups into social hierarchies (Hackett et al., 2020; Williams & Medlock, 2017). Furthermore, RD reinforces stereotypes and attitudes against stigmatized racial groups, marginalizing individuals from societal resources and opportunities and contributing to the perpetuation of racism (Williams et al., 2019). Racism, a facet of RD and often used interchangeably with RD throughout the literature (Alhusen et al., 2016), implies an ideology of racial superiority/inferiority and the hierarchization of racial groups (Pager & Shepherd, 2008). Both individuals and social institutions can reproduce it, which can be persistent through governmental policies (Williams, 2018). Individuals exposed to RD may bear the stigma burden of their racial group, raising chronic vigilance, rumination of racial incidents, and anticipatory concerns over potential exposures (Hicken et al., 2018; Lewis et al., 2015).

New evidence suggests that RD is becoming more frequent in society. Individuals have been reporting more experiences of RD manifested through racial slurs (Huang & Cornell, 2019), hate crimes (Myers & Lantz, 2020), and governmental policies perceived as discriminatory by targeted groups (Williams & Medlock, 2017). Political shifts in several countries are also a factor that has been associated with more overt expressions of offensive language and attitudes toward minorities (da Silva & Larkins, 2019; Williams & Medlock, 2017). Additionally, social media represents a growing setting of exposure to RD (Criss et al., 2021). Thus, the pervasiveness of RD in society makes RD a constant source of psychological stress with detrimental implications for well-being (Vargas et al., 2020).

Evidence suggests that RD may contribute to a sense of diminished self-worth (Benner et al., 2018), loneliness (Priest et al., 2014), and hopelessness due to the reduced opportunities for upward mobility (Clark et al., 1999). Also, perceived RD may elicit cognitive appraisals of threat, as individuals become a deliberate target to others because of their inheritable characteristics (Dion, 2002). As a result, meta-analyses found associations between RD and lower life satisfaction (Harris et al., 2018), poorer self-rated health (Schmitt et al., 2014), and mental disorders such as depression (Britt-Spells et al., 2018), anxiety (Paradies et al., 2015), and posttraumatic stress disorder (Kirkinis et al., 2021). Notably, mental disorders are considered risk factors for suicide (Brådvik, 2018).

Suicide is among the twenty leading causes of death worldwide (around 700,000 suicides each year), with one person taking their own life every 40 s (WHO, 2019). Death by suicide represents an individual and a social tragedy with a significant psychological and socioeconomic impact on families, friends, and entire communities (Cerel et al., 2019). It is a significant public health problem and has been the focus of growing attention in social and health sciences and public awareness (Turecki et al., 2019). Although suicide rates declined globally in the 20th century, they increased by 7% in the late 20th/early 21st century (Naghavi & Collaborators, 2019) and by 30% in the U.S between 2000 and 2016 (Hedegaard et al., 2018). Racial groups may differ in suicide rates, but racial minorities and marginalized racial groups appear to be at high risk. Research findings report a higher suicide risk among migrants of ethnic minorities in Europe (Forte et al., 2018), indigenous groups in Oceania (Dickson et al., 2019; Hatcher, 2016) and South America (Troya et al., 2022), and increasing suicide rates for Blacks and Native Americans in the U.S (Lindsey et al., 2019; Shain, 2019). Notably, increasing rates of suicide among young individuals from minority groups have also been observed across different societies (Dickson et al., 2019; Forte et al., 2018), elevating suicide as the second leading cause of death among Native American youth and the third among African American youth (Heron, 2016; Shaughnessy et al., 2004).

Given that certain groups are more exposed to RD and RD has negative consequences for psychological well-being, studies began to investigate whether RD is associated with suicidal ideation (SI) and with a history of suicide attempt (SA). Several studies reported a positive association between RD and SI or SA across racially diverse groups in the U.S. (Chao et al., 2012; Choi et al., 2020; Galán et al., 2021; Perez-Rodriguez et al., 2014; Walls et al., 2007), among Blacks in Brazil (Santana et al., 2007) and Canada (Okoye & Saewyc, 2021), Aboriginals in Australia (Priest et al., 2011), Māori in New Zealand (Williams et al., 2018), and ethnic minorities in China (Pan & Spittal, 2013). As of yet, the only published meta-analysis investigating the association between RD and suicidality reported an association between the two phenomena (*r* = −0.16; Paradies et al., 2015). However, this meta-analysis compiled studies published in the literature until 2013, and only ten studies were available. Since then, more evidence has emerged, part of which reports mixed or null findings (Hong, 2019; Lacey et al., 2021; Layland et al., 2020; Oh & Nicholson, 2021; Polanco-Roman et al., 2021; Taliaferro et al., 2020), suggesting that the question whether RD is associated with suicidality needs to be further addressed in the literature. This highlights the importance of an updated meta-analysis investigating the magnitude of the relationship between RD and suicidality.

In the present article, we aimed to meta-analytically review the literature and investigate the magnitude of the relationship between racial discrimination (whether based on race or its related classifiers such as ethnicity and nationality) and suicidality. We considered both dimensional aspects of suicidality separately in our study: suicidal ideation and suicide attempt, and we conducted separate analyses to investigate the impact of RD on both. We defined SI as thoughts/wishes about ending one's own life, which may include suicidal plans or not (Turecki et al., 2019), and SA as an act of intention to take one's own life deliberately. As several studies indicate that RD and suicide are increasing phenomena among marginalized racial groups and racial minorities, we hypothesize that our meta-analytical review will find a statistically significant effect of RD on suicidality.

## 2 Methods

### 2.1 Search strategy

We retrieved articles for this meta-analysis from Embase, PubMed, PsycINFO, and Scopus with a publication date prior to September 25, 2021. Details of the protocol for this review were preregistered on the International Prospective Register of Systematic Reviews (PROSPERO) with registration number CRD42021268089. We used a comprehensive list of terms to conduct the systematic search (see supplementary material, Part 1). Two reviewers (BMC and JY) screened the initial 100 records together and the following 250 records independently. The independent search of the 250 records resulted in an excellent interrater agreement (kappa = 0.8). Each of the two reviewers (BMC and JY) screened half of the remaining records. Additionally, we hand-searched references of the eligible articles and other reviews to identify potential missing studies in our systematic search. The systematic search was conducted according to the updated guidelines of the Preferred Reporting Items for Systematic Reviews and Meta-Analyses (PRISMA) (Page et al., 2021)

### 2.2 Inclusion and exclusion criteria

Articles eligible for this meta-analysis were those that investigated whether racial discrimination in any form (e.g., racism, racial bullying, racial microaggression, discrimination due to ethnicity or nationality) is associated with suicidality, i.e., suicidal ideation and/or a history of a suicide attempt. All articles had to be written in English and published in peer-reviewed journals prior to September 25, 2021 (our final search date). For studies that measured different dimensions of RD on suicidality, we opted to consider the more direct forms of RD experiences (e.g., overt RD instead of subtle RD, interpersonal RD instead of vicarious RD). We excluded articles that a) investigated types of discrimination other than RD; and b) investigated multiple or combined types of discrimination where it is not possible to disentangle RD from other types of discrimination (e.g., gendered RD). Articles that only investigated death-wish without suicidal thoughts or non-suicidal self-injury/self-harm thoughts and behaviors were also excluded.

### 2.3 Data extraction and quality assessment

One reviewer (BMC) extracted data from the selected articles, and another reviewer (CH) verified the extraction accuracy. For all eligible articles, we extracted the following data: first author and year of publication; sample size; sex/gender (percentage of female-identifying individuals); age range (with mean age and SD if available); population (city, region, or country); race/ethnicity; sample recruitment (community sample, students, or larger study); study design; suicidality assessment and time frame of investigation; RD assessment, categorization, and time frame of exposure; outcomes and findings (see Table 1S in supplementary material, Part 2, for complete data extraction). Two raters (BMC and JY) independently assessed the quality of studies using the NIH Quality Assessment Tool for Observational Cohort and Cross-sectional Studies (NIH, 2013). Studies were rated as good, fair, or poor. Ratings were discussed between the reviewers. Disagreements on the quality of the studies were resolved with the help of a third rater (CH).

### 2.4 Potential moderators

We coded several study characteristics to investigate potential moderators of the effect of RD on suicidality: a) RD and suicidality measures (dichotomous items versus Likert item(s)/non-validated scale versus validated scale); b) sample origin (U.S. versus non-U.S., given that we expect that the majority of eligible studies will be from the U.S.); c) study design (cross-sectional versus longitudinal); d) mean age; e) age (under 25 years of age versus all other ages); f) race (Black versus all other races, or native peoples versus all other races); g) quality assessment ratings; h) RD time frame assessment (>12 months/lifetime versus ≤12 months); i) SI time frame assessment (>12 months/lifetime versus ≤12 months); j) RD categorization, i.e., we categorized studies according to how they assessed RD: occurrence of exposure to RD (yes or no), frequency (how often), appraisal (rated stressfulness or reaction), or a combination of frequency/appraisal.

### 2.5 Statistical analysis

We used the R package meta for the analyses (Schwarzer, 2010). The effect of RD experiences on suicidality was determined by pooling effect sizes of the relationship between RD and SI and between RD and having a history of SA without any covariates. We standardized all effect sizes to Pearson's correlation coefficient (*r*). A positive correlation would indicate a positive relationship between RD and SI or between RD and SA. We contacted the authors for papers that did not provide sufficient data for calculating the effect size and requested additional data. Of the 14 studies whose authors we contacted, we obtained nine positive responses with the requested data. Authors from three studies did not respond to our e-mails, and authors from two studies responded that they no longer had access to data.

We verified heterogeneity between studies (assessed with the Q index) using a random-effects model. Also, in addition to visually inspecting the funnel plots for SI and SA for asymmetry (a sign of publication bias), we performed two rank tests: Kendall's tau and Egger's test. In case of an indication of publication bias, we used a trim and fill procedure to correct for bias of the missing studies. If direct findings of the effects of RD on SI and SA were statistically significant, we performed two fail-safe tests to assess the robustness of the results: Rosenthal's and Rosenberg's methods. Lastly, to test if the characteristics of the studies were related to differences in the effect sizes for SI and SA, we conducted moderation analyses with a meta-regression approach by fitting mixed-effect models.

## 3 Results

### 3.1 Literature search

**Fig. 1:**
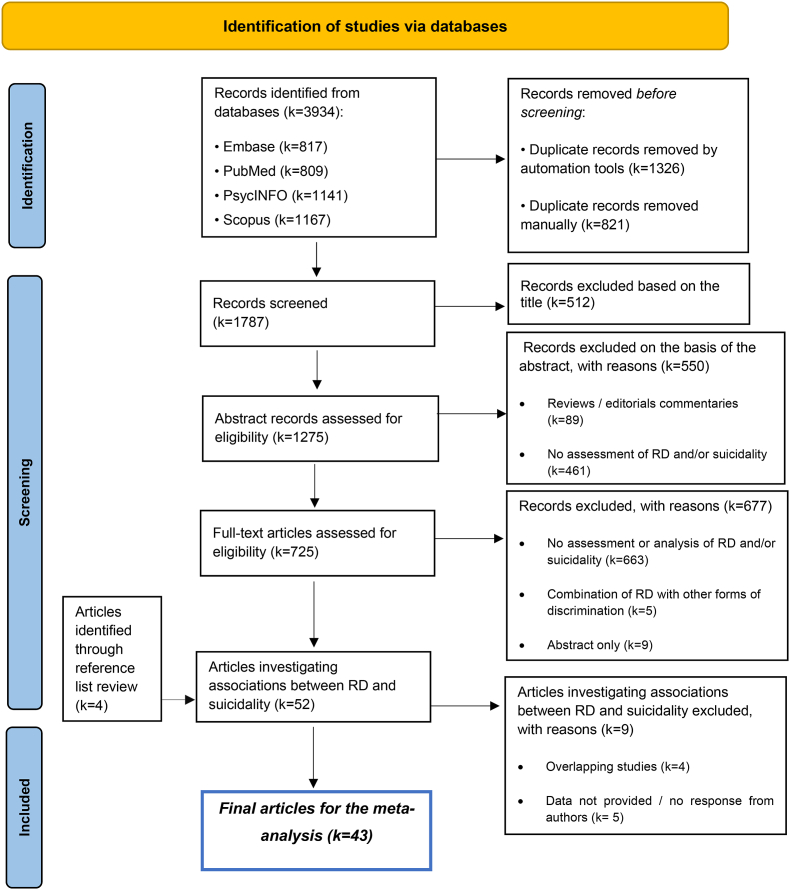
Flow diagram of the article selection.

The complete study identification process is visualized in the PRISMA diagram (Fig. 1). Our search strategy identified 3934 records through Embase, PubMed, PsycINFO, and Scopus, of which 2147 were duplicates. Of the 1787 remaining records, we excluded 512 records based on their titles, resulting in 1275 records for screening. Further, we assessed the abstracts of the 1275 records and excluded 550 based on their abstracts. The remaining 725 full-text articles were assessed, of which 677 were excluded (see Fig. 1 for reasons). Forty-eight articles were identified through the systematic search, and four articles identified through reference list review investigated the association between RD and suicidality. Of these 52 articles, we removed nine articles from the meta-analysis due to overlapping samples (k = 4), which would provide us with repeated effect sizes, or because we could not obtain effect sizes (k = 5). Lastly, we pooled effect sizes from 43 studies for the quantitative synthesis, resulting in 39 effect sizes for the association between RD and SI and 15 effect sizes for the association between RD and having a history of SA.

### 3.2 Characteristics of included studies

We provide the complete data extraction of all 52 articles investigating the association between RD and suicidality in Table 1S. In Table 1, we briefly describe the 43 studies available for the quantitative synthesis and the effect sizes pooled for the relationship between RD and SI and between RD and SA. The 43 studies contained 62,349 individuals, of which 58,629 individuals provided data for the relationship between RD and SI and 30,088 individuals for the relationship between RD and a history of SA. Most of the studies were from the U.S. (k = 36), were predominantly composed of females (k = 27), individuals under 25 years of age (k = 23), and had racially/ethnically diverse samples (k = 27, for two or more races/ethnicities).

**Table 1:** Selected characteristics of studies examining the association between racial discrimination and suicidality.

Reference	Sample size	Female, %	Age range (mean, SD)	Population	Race/ethnicity	Effect size (SI or SA)	Analytic stratification*	Quality assessment
Freedenthal et al., 2004	311	56.9%	12-20 (15.6, 1.6)	Southwestern U.S.	Native American	0.15 (SA)	RD measure: dichotomous; RD categorization: occurrence of exposure	Fair
Yoder et al., 2006	201	46%	9-16 (12.12, 1.44)	Reservations in Midwestern U.S.	Native American	0.20 (SI)	SI measure: dichotomous; RD measure: Likert item(s); RD categorization: frequency	Good
Santana et al. (2007)	973	68.9%	10-21 (NA)	Salvador, BA, Brazil	Black & non-Black Brazilians	0.14 (SI)	SI measure: dichotomous; RD measure: dichotomous; RD categorization: occurrence of exposure	Good
Walls et al. (2007)	721	50%	10-13 (11.1, NR)	Midwest U.S. & Canada	Native American	0.14 (SI)	SI measure: dichotomous; RD measure: Likert item(s); RD categorization: frequency	Good
Hwang & Goto, 2008	186	63.9%	NR (NR)	Rocky Mountains, U.S.	Asian American & Latino	0.146 (SI)	SI measure: validated scale; RD measure: validated scale; RD categorization: frequency	Good
Castle et al. (2011)	234	NR	18-24 (NR)	Northeastern U.S.	African-American	0.005 (SI) 0.005 (SA)	SI measure: validated scale; RD measure: validated scale; RD categorization: appraisal	Fair
Gomez et al., 2011	969	68%	18-25 (18.8, 1.3)	Northeastern U.S.	Asian, Latino, Black & White	0.114 (SA)	RD measure: validated scale; RD categorization: appraisal/frequency	Good
Hightow-Weidman et al. (2011)	348	0%	NR (20.4, NR)	Bronx, NY; Chapel Hill, NC; Chicago, IL; Detroit, MI; Houston, TX; Los Angeles, CA; Oakland, CA; Rochester, NY	African-American, Latino, and multiracial	0.0988 (SI) 0.0467 (SA)	SI measure: dichotomous; RD measure: Likert item(s); RD categorization: occurrence of exposure	Fair
Priest et al. (2011)	345	53%	16-20 (18.27, 1.06)	Northern Territory, Australia	Australian Aboriginal	0.23 (SI)	SI measure: Likert item(s); RD measure: dichotomous; RD categorization: occurrence of exposure	Good
Chao et al. (2012)	1555	63%	18-53 (23.14, 5.78)	Midwestern U.S.	African-American	0.30 (SI)	SI measure: Likert item(s); RD measure: Likert item(s); RD categorization: appraisal	Good
Luke et al., 2013	172	56.4%	19 (12–26, 4)	Melbourne, Australia	Australian Aboriginal	0.22 (SI) 0.31 (SA)	SI measure: dichotomous; RD measure: dichotomous RD categorization: occurrence of exposure	Fair
Pan and Spittal (2013)	8182	51.40%	13-15 (NR)	Chinese cities of Beijing, Hangzhou, Wuhan & Urumqi	Chinese (ethnicity not specified)	0.21 (SI)	SI measure: dichotomous; RD measure: dichotomous; RD categorization: occurrence of exposure	Fair
Polanco-Roman & Miranda (2013)	143	80%	18-25 (18.6, 1.19)	Northeastern U.S.	Asian, Black, Latino, White	0.08 (SI)	SI measure: validated scale; RD measure: validated scale; RD categorization: appraisal/frequency	Good
Thoma & Huebner, 2013	276	33%	14-19 (17.45, 1.36)	Indianapolis, IN, Oakland, CA, Boston, MA, Philadelphia, PA	African-American, African-American mixed	0.12 (SI)	SI measure: dichotomous; RD measure: validated scale; RD categorization: appraisal/frequency	Fair
Perez-Rodriguez et al. (2014)	6359	49.1%	18-65+ (NR)	U.S. (several areas)	Hispanic	0.051 (SI)0.020 (SA)	SI measure: dichotomous; RD measure: dichotomous; RD categorization: occurrence of exposure	Fair
Walker et al., 2014	236	48.6%	18-59 (36.46, 12.47)	One southeastern community in the U.S.	African-American	0.13 (SI)	SI measure: validated scale; RD measure: validated scale; RD categorization: appraisal/frequency	Fair
Brockie et al., 2015	285 for SI, 279 for SA	51%	15-24 (19.25, 2.9)	One reservation (unidentified) in the U.S.	Native American	0.155 (SI) 0.130 (SA)	Si measure: dichotomous; RD measure: Likert item(s); RD categorization: frequency	Good
Chesin & Jeglic, 2015	118	86%	≅20	Northeastern U.S.	African American, White & other	0.20 (SI)	SI measure: validated scale; RD measure: validated scale; RD categorization: appraisal/frequency	Good
O'Keefe et al., 2015	405	61.4%	18-48 (19.65, NR)	Midwest U.S.	African-American, Asian American, Hispanic & Native American	0.13 (SI)	SI measure: validated scale; RD measure: validated scale; RD categorization: appraisal/frequency	Good
Gattis & Larson, 2016	89	52%	16-24 (20.60, 2.06)	Milwaukee, WI	African-American	0.31 (SI)	SI measure: dichotomous; RD measure: validated scale; RD categorization: appraisal/frequency	Good
Sutter & Perrin, 2016	200	53%	NR (29.5, 9.93)	U.S. (several areas)	African American, Latino, Asian, Native American & mixed race	0.14 (SI)	SI measure: validated scale; RD measure: validated scale; RD categorization: frequency	Good
Arshanapally et al. (2018)	796	50%	13-32 (17.9, NR)	Missouri, MS	African-American	0.2799 (SI) 0.1795 (SA)	SI measure: dichotomous; RD measure: validated scale; RD categorization: appraisal/frequency	Fair
Hong et al. (2018)	289	61.2%	18-25 (20.47, 1.83)	Southwestern U.S.	African-American, Asian American, Hispanic	0.24 (SI)	SI measure: validated scale; RD measure: validated scale; RD categorization: frequency	Fair
Kwon & Han, 2018	1916	49%	NR (37.35, 14.64)	U.S. (several areas)	Latino	0.1949 (SI)	SI measure: dichotomous; RD measure: Likert item(s); RD categorization: frequency	Fair
Li et al., 2018	3123	57.7%	60+ (72.3, 8.3)	Chicago, IL	American Chinese	0.1543 (SI)	SI measure: validated scale; RD measure: validated scale; RD categorization: appraisal/frequency	Good
Williams et al. (2018)	1623	54.4%	12-19 (NR)	New Zealand	Maori	0.21 (SA)	RD measure: Likert item(s); RD categorization: occurrence of exposure	Good
Hong (2019)	292	56.5%	Over 19 (42.6, 10.3)	Seoul and nearby areas, South Korea	Korean Chinese	0.20 (SI)	SI measure: dichotomous; RD measure: dichotomous; RD categorization: occurrence of exposure	Fair
Polanco-Roman et al. (2019)	1344	72%	18-29 (19.88, 2.25)	Northeastern U.S.	African-American, Hispanic/Latino, White, Asian & other	0.09 (SI)	SI measure: validated scale; RD measure: validated scale; RD categorization: frequency	Good
Choi et al. (2020)	786	56.35%	11-19 (15, 1.91)	Chicago area, IL	Asian American (Korean & Filipino)	0.26 (SI)	Si measure: dichotomous; RD measure: Likert item(s); RD categorization: frequency	Good
Edwards et al., 2020	400	54.9%	12-18 (13.88, 1.30)	Great Plains, U.S.	American Indian & Alaska Native	0.18 (SI)	SI: dichotomous; RD measure: dichotomous; RD categorization: occurrence of exposure	Fair
Layland et al. (2020)	817	50.8%	18-68 (39.1, 12.3)	U.S. (several areas)	Non-Hispanic Black and Hispanic	0.08 (SA)	RD measure: validated scale; RD categorization: appraisal/frequency	Good
Taliaferro et al. (2020)	435	56%	18-26 (NR)	Midwestern & Southeastern, U.S.	Asian, European, Hispanic/Latino, African, Middle Eastern, and other	0.24 (SI)	SI measure: Likert item(s); RD measure: validated scale; RD categorization: frequency	Good
Argabright et al., 2021	11235	47.7%	9-10 (10.9, 0.64)	U.S. (several areas)	White, Black, Asian, American Indian, Native Hawaiian & other	0.32 (SI)	SI measure: dichotomous; RD measure: Likert item(s); RD categorization: occurrence of exposure	Good
Galán et al. (2021)	3650	53.7%	NR (15.7, 1.3)	Pittsburgh, PA	White, Black, or African American, Asian, American Indian, or Alaska Native, Native Hawaiian or Other Pacific Islander, and other	0.071 (SI)	SI measure: dichotomous; RD measure: dichotomous; RD categorization: occurrence of exposure	Fair
Goodwill et al. (2021)	1271	0%	18-93 (43.53, 16.18)	U.S. (several areas)	African-American	0.091 (SI)	SI measure: dichotomous; RD measure: validated scale; RD categorization: frequency	Good
Lacey et al. (2021)	3277	100%	NR (42.5, NR)	U.S. (several areas)	African American & Caribbean Black	0.04 (SI)	SI measure: dichotomous; RD measure: validated scale; RD categorization: frequency	Good
Mallory & Russel, 2021	585	49%	15-24 (19, 1.79)	One city in the Northeast and one city in the Southwest, U.S.	Black, Latino, Asian, Multi-racial, Native American, Hawaiian & Pacific Islander	0.21 (SI)	SI measure: validated scale; RD measure: validated scale; RD categorization: appraisal/frequency	Good
Oh and Nicholson (2021)	5191	NR	18+ (NR)	U.S. (several areas)	African Americans & Caribbean Black Americans	0.01 (SI) 0.008 (SA)	SI measure: dichotomous; RD measure: Likert item(s); RD categorization: frequency	Fair
Okoye and Saewyc (2021)	535 for SI, 533 for SA	48%	NR (15.1, NR)	British Columbia, Canada	African Canadians	0.17 (SI) 0.20 (SA)	SI measure: dichotomous; RD measure: dichotomous; RD categorization: occurrence of exposure	Fair
Polanco-Roman et al. (2021)	747	61%	18-29 (19.84, 2.22)	Northeast U.S.	Asian, Hispanic, Black & other	0.08 (SI) 0.04 (SA)	SI measure: validated scale; RD measure: validated scale; RD categorization: frequency	Good
Wang et al., 2021	960	76%	NR (44.47, 15.65)	U.S. (several areas)	Non-Hispanic Black, non-Hispanic American Indian/Alaska Native, non-Hispanic Asian/Native Hawaiian/Other Pacific Islander, and Hispanic	0.06 (SI) 0.02 (SA)	SI measure: dichotomous; RD measure: Likert item(s); RD categorization: frequency	Good
Madubata et al. (2022)	157	49%	14-17 (15.1, 0.53)	Southeast U.S.	African-American & Latinx	0.14 (SI)	SI measure: validated scale; RD measure: Likert item(s); RD categorization: frequency	Good
Zimmerman & Miller-Smith (2022)	1147	50.31%	9-12 (10.61, 1.54)	Chicago, IL	Hispanic, African-American, White, Asian, Pacific Islander, Native American, mixed-race	0.1067 (SI)	SI measure: dichotomous; RD measure: Likert item(s); RD categorization: occurrence of exposure	Good

***Note****.* NR: Not reported; SI: Suicidal ideation; SA: Suicide attempt. * All SA measures were dichotomous.

### 3.3 Measures of RD and suicidality across studies

The literature presents a significant variability of approaches to measure RD and SI (see Table 1, analytical stratification). Of the 43 available studies for the qualitative analysis, most studies used a single dichotomous item (participants were coded for “yes” or “no” answers) or Likert item(s)/non-validated instruments to assess RD experiences (k = 23). Twenty studies used 11 different validated instruments to measure RD. Except for appraisal (k = 2), RD categorization was well distributed across eligible articles: occurrence of RD exposure (k = 14), frequency (k = 13), and appraisal/frequency (k = 14).

Similarly, there was a large variability of validated instruments to assess SI: ten different validated instruments were applied across 13 studies. Most studies measuring SI used a dichotomous item (responses were coded “yes” or “no” if participants presented SI, k = 22) or Likert item(s)/non-validated instruments (k = 4). For SA, eligible studies used a dichotomous item to code whether participants have ever tried to take their own life.

Furthermore, we observed that time frame assessments of RD and SI differed across studies (from current to lifetime). Thirty studies queried lifetime RD or in a period longer than the past 12 months, whereas 13 studies queried RD in the past year or less. As for SI, 14 studies investigated lifetime SI or in a period longer than the past 12 months, and 25 studies investigated it in the past year. Except for one study, all studies investigating SA queried lifetime SA. We offer a detailed assessment of the time frame in Table 1S.

### 3.4 Effect of racial discrimination on suicidality

**Fig. 2:**
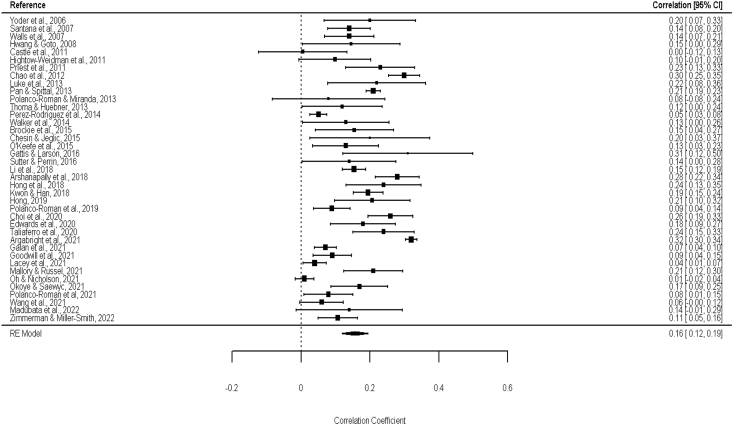
Forest plot with Pearson's Correlation coefficient (r) between racial discrimination and suicidal ideation.

**Fig. 3:**
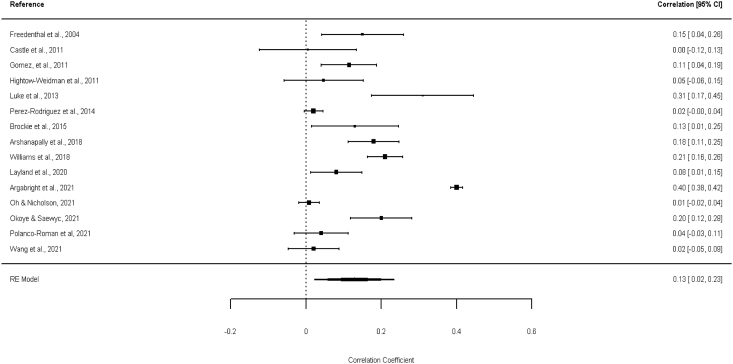
Forest plot with Pearson's Correlation coefficient (r) between racial discrimination and suicide attempt.

**Fig. 4:**
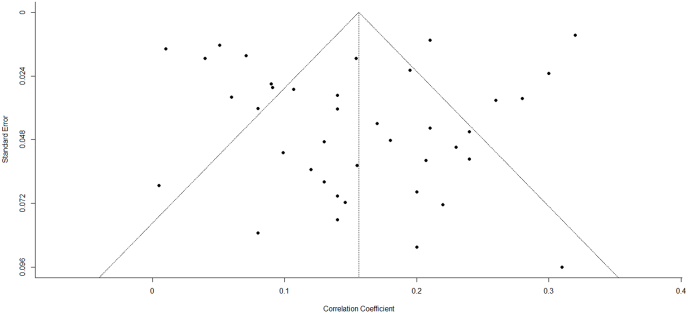
Funnel plot with Pearson's correlation coefficient (*r*) between racial discrimination and suicidal ideation.

**Fig. 5:**
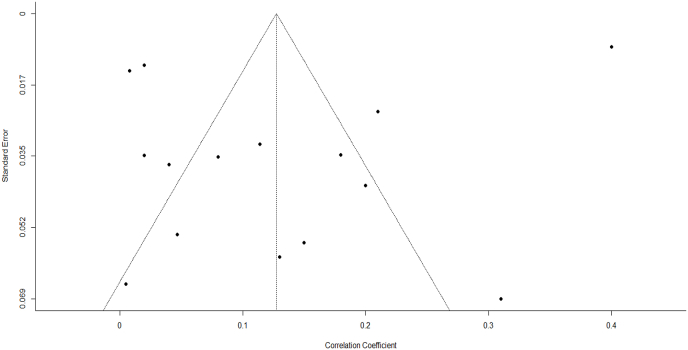
Funnel plot with Pearson's correlation coefficient (*r*) between racial discrimination and suicide attempt.

Fig. 2 shows the main results for the analysis on RD and SI, and Fig. 3 shows the main results for the analysis on RD and having a history of SA. The pooled correlation between RD and SI was 0.16 (*p* < 0.0001), and the pooled correlation between RD and SA was 0.13 (*p* = 0.018). The heterogeneity between studies for both SI and SA was high: I^2^ = 94.91%, *p* < 0.0001 and I^2^ = 98.72%, *p* < 0.0001, respectively. Visual inspection of the funnel plots did not suggest asymmetry (Fig. 4, Fig. 5), which was confirmed by the Kendall's tau correlation coefficient (0.13, *p* = 0.21 for SI; 0.16, *p* = 0.43 for SA) and by the Egger's test (*t* = −0.93, *p* = 0.35 for SI; *t* = −1.58, *p* = 0.13 for SA). The safe-fail tests confirmed the robustness of the results (Rosenberg fail-safe n = 18,362 for SI and n = 6040 for SA. Rosenthal fail-safe n = 14,442 for SI and n = 2887 for SA).

### 3.5 Effect of potential moderators of the effect of racial discrimination on suicidality

Table 2, Table 3 show the results of the moderation analyses. Moderators were age, sample origin, RD and suicidality measures, study design, race, quality assessment ratings, RD time frame assessment, and SI time frame assessment. The only moderator associated with the effect of RD on SI was the SI time frame assessment (*p* = 0.01). We found a more significant effect size for studies that assessed SI in the past year than those that measured lifetime SI or in a period longer than 12 months (*r* = 0.18 versus *r* = 0.11). We also found a trend of significance between RD time frame assessment (past 12 months) and SI (*p* = 0.09). None of the moderators was associated with the effect of RD on SA. Although we observed a trend of significance between having a history of SA and younger mean age (*p* = 0.07) and between SA and non-US samples (*p* = 0.08), these relationships did not reach statistical significance levels.

**Table 2:** Effect of racial discrimination on suicide ideation and moderation analysis.

	k	Pearson's *r*	95% CI	*p*
Overall outcome	39	0.16	0.12 to 0.19	**<0.0001**
Moderation Analyses				
Mean age	30	−0.001	−0.003 to 0.001	0.28
Age	29	−0.02	−0.08 to 0.03	0.42^a^
Sample origin	39	−0.04	−0.12 to 0.26	0.24^a^
RD measure	39	−0.008	−0.04 to 0.02	0.63^a^
RD categorization	39	−0.0005	−0.02 to 0.02	0.96^a^
SI measures	38	−0.004	−0.03 to 0.02	0.76^a^
Study design	39	−0.009	−0.08 to 0.06	0.80^a^
Black vs other	39	0.008	−0.06 to 0.07	0.80^a^
Native vs other	39	0.02	−0.04 to 0.10	0.45^a^
Quality assessment	39	0.02	−0.03 to 0.08	0.39^a^
RD time frame	39	0.05	−0.008 to 0.10	0.09^a^
SI time frame	39	0.07	0.015 to 0.12	**0.01**^**a**^

*Note.*^a^ P-value indicates whether the effect size of subgroups differs significantly. Categorical moderators: age (under 25 years of age versus all other ages); Sample origin (U.S. versus non-U.S.); RD measure (dichotomous items versus Likert items/non-validated scale versus validated scale); RD categorization (occurrence of RD versus frequency versus appraisal versus appraisal/frequency); SI measures (dichotomous items versus Likert items/non-validated scale versus validated scale; Study design (cross-sectional versus longitudinal); Black versus all other races; Native peoples versus all other races; Quality assessment ratings; RD time frame assessment (>12 months/lifetime versus ≤12 months), SI time frame assessment (>12 months/lifetime versus ≤12 months).

**Table 3:** Effect of racial discrimination on suicide attempt and moderation analysis.

	k	Pearson's *r*	95% CI	*p*
Overall outcome	15	0.13	0.02 to 0.23	**0.018**
Moderation Analyses				
Mean age	10	−0.008	−0.01 to 0.52	0.07
Age	12	−0.04	−0.18 to 0.10	0.58^a^
Sample origin	15	−0.13	−0.27 to 0.01	0.08^a^
RD measures	15	−0.03	−0.11 to 0.04	0.36^a^
RD categorization	15	−0.03	−0.07 to 0.016	0.2^a^
Study design	15	−0.11	−0.35 to 0.12	0.35^a^
Black vs other	15	0.009	−0.15 to 0.16	0.91^a^
Native vs other	15	0.09	−0.04 to 0.23	0.18^a^
Quality assessment	15	0.03	−0.08 to 0.16	0.57^a^

*Note.*^a^ P-value indicates whether the effect size of subgroups differs significantly. Categorical moderators: age (under 25 years of age versus all other ages); Sample origin (U.S. versus non-U.S.); RD measures (dichotomous items versus Likert items/non-validated scale versus validated scale); RD categorization (occurrence of RD versus frequency versus appraisal versus appraisal/frequency); Study design (cross-sectional versus longitudinal); Black versus all other races; Native peoples versus all other races; Quality assessment ratings.

## 4 Discussion

In the present meta-analysis, we investigated whether racial discrimination is associated with suicidal ideation and having a history of suicide attempt. We located 52 studies investigating the association between RD and suicidality, from which 43 studies were eligible for the quantitative synthesis. Our meta-analysis indicates that RD has a small but statistically significant effect on SI (*r* = 0.16, *p* < 0.0001) and on SA (*r* = 0.13, *p* = 0.018). Also, we identified that the SI time frame measure has a significant moderating effect on SI outcomes. Studies that measured SI in the past year had a larger effect size than studies that measured lifetime SI or in a period longer than 12 months (*r* = 0.18 versus *r* = 0.11, *p* = 0.01). This suggests a pattern that SI should be assessed in a more time-limited way when investigating to what extent SI is affected by discriminatory experiences. Assessing lifetime SI may be subjected to memory bias, and perception of SI is likely more salient when assessing individuals using a time-limited and more recent time frame approach. Apart from the SI time frame, the effect of RD on suicidality could not be explained by other studies’ characteristics, as none of the other analyzed moderators reached statistical significance levels.

Furthermore, we found no indication of publication bias in our approach to investigating the effect of RD on SI and SA after performing two different tests (Egger's test and Kendall's tau). Fail-safe tests confirmed the robustness of our results (Rosenberg's and Rosenthal's methods). Our findings suggest that suicidality is a phenomenon that race-related discriminatory experiences may influence. Thus, we confirmed our hypothesis that individuals who experience discrimination based on race might develop more suicidal thoughts and an increased likelihood of attempting suicide.

Our results align with findings reported by a previous meta-analysis that conducted a comprehensive investigation of the effects of RD on multiple health problems, including suicidality (Paradies et al., 2015). However, this meta-analysis reported findings on ten available studies of RD and suicidality, whereas ours found 43 studies, thus, offering an updated status of the literature. Our meta-analytical study is the first to consider an exclusive investigation of RD and suicidality and to perform two separate analyses measuring the effects of RD both on SI and on SA. Our study adds to the growing body of literature indicating that RD is pernicious to well-being and may be a driver of health disparities in society, as groups exposed to RD may exhibit accentuated decline in mental health parameters (Coimbra et al., 2020; Kairuz et al., 2021; Metzner et al., 2022; Williams & Williams-Morris, 2000). As such, more awareness of the issue of RD and the appropriate development of more effective intervention strategies to tackle its effects should be a concern among health workers, researchers, educators, and policymakers.

The majority of the studies in our meta-analysis enrolled samples of adolescents and young adults. Research findings suggest the plausibility that RD is increasingly influential in youth suicide deaths (Al-Mateen & Rogers, 2018). Young individuals may be more aware of racial tensions in society nowadays (Sewell et al., 2016) and more vulnerable to RD exposure on social media (Criss et al., 2021). As the racial composition is shifting in the U.S. and more individuals identify with a non-White racial/ethnical group, young racial minority adults will likely comprise a growing percentage of the nation's suicides (Castle et al., 2011). Although this highlights the relevance of studies on suicidality among the youth, we did not find age to have a moderating effect on the association of RD with SI and SA. This suggests that race-based discrimination may affect suicidality regardless of age and that further studies should not overlook samples with older individuals.

Although most of the studies in our review are from the U.S., we retrieved eligible studies from Canada, Brazil, China, South Korea, Australia, and New Zealand. The current state of the evidence does not show distinctions between U.S. and non-U.S. studies, suggesting that RD may affect suicidality independently of cross-national differences in social dynamics. However, the underrepresentation of samples from developing nations is worth mentioning, as it limits conclusions to high-income countries. Researchers recommend further expanding knowledge on RD and suicide in low- and middle-income countries (Glenn et al., 2020; Olusanya, 2021). This expansion could further our understanding of whether the effect of RD on suicidality that we found in our meta-analysis is indeed a worldwide phenomenon.

Our study has some limitations. First, the complexity of measuring RD is acknowledged in the literature (Williams, 2016). Challenges may be related to the accuracy of measurements of elements related to racially discriminatory experiences, e.g., appraisal, frequency, severity, stress, and time frame of exposure. Future studies should address these issues (Williams et al., 2019). Since we performed a meta-analysis, we are limited to the instruments used across studies. We attempted to categorize RD measures to investigate some of these issues, but we did not find a moderating effect on suicidality.

Moreover, many studies in our article selection use a single dichotomous item or Likert item to assess the occurrence, frequency, or appraisal of RD and/or SI. Such measures might not capture essential nuances of adverse experiences and perceptions related to RD and suicidality. We urge future studies to include validated instruments in their assessments.

A further limitation may be related to our moderators. The effect sizes used in this meta-analysis were primarily pooled from studies with racially diverse samples of both females and males. Various statistical models with the inclusion of multiple covariates were used in these studies to investigate the effect of RD on suicidality. Although we successfully obtained a large number of effect sizes of the correlation between RD and suicidality, our approach with the selected moderators may have been limited to capturing differences in SI and SA according to race because we could not obtain effect sizes for specific groups within studies. Thus, as some eligible articles in our review investigated suicidality only in Blacks (k = 10) and in Native peoples (k = 7), we compared the effect sizes from these studies with effect sizes from studies with multiple races or with other single races. However, these studies with multiple racial groups also generally contained Blacks and Native peoples, which may have yielded our approach too imprecise; and it is important to note that most studies in our article selection with individuals from multiple racial groups did not report the results of an investigation separate by race. The exceptions are Gomez et al. (2011), who reported increased SA among Latinos and non-American Whites compared to Asians, Whites, and Blacks; Madubata et al. (2022), who found increased SI for Blacks compared to Latinx; and Hwang & Goto (2008), who reported no differences between Latinos and Asian Americans. Furthermore, by the time we completed our systematic search, there was no published evidence on the effects of race-based discrimination on suicidality among Asian Americans during the Covid-19 pandemic. As Asian Americans have reported increased experiences of RD in the Covid-19 emergence with adverse mental health consequences (Lee et al., 2022), the literature may benefit from studies that target this specific group in suicidality studies.

Similarly, we could not disentangle differential effects regarding sex/gender and investigate it as a moderator. Although some papers in our review found sex differences in the associations between RD and suicidality in their models (Polanco-Roman et al., 2019; Zimmerman & Miller-Smith, 2022), other papers reported no differences (Arshanapally et al., 2018; Chao et al., 2012). However, the vast majority of papers did not consider sex differences, and only three studies enrolled same-sex samples (Goodwill et al., 2021; Hightow-Weidman et al., 2011; Lacey et al., 2021), invalidating any comparative analysis. RD may affect males' and females' expression of suicidality in different ways. However, our study could not contribute to more specific knowledge on sex/gender differences due to the limited data.

In summary, our study successfully investigated RD and suicidality across 43 studies from seven countries, providing a quantitative synthesis of the effect of RD on suicidality. We found that RD has a small but statistically significant effect on SI and SA. Thus, among other factors that may influence suicidality, RD may be a social factor that increases the risk of developing suicidal thoughts and attempting suicide. These findings may be relevant to researchers in the field of social sciences and mental health. Lastly, our meta-analysis is the first to investigate the effects of RD in explaining SI and SA separately. Furthermore, only studies that investigated racial discrimination apart from any other form of discrimination were included. As emerging evidence indicates that racial discrimination is an increasing social phenomenon, more prevention and intervention efforts are needed to attenuate its effect on suicidality.

## Ethical statement

Hereby, I Bruno Messina Coimbra consciously assure that for the manuscript *“Meta-analysis of the effect of racial discrimination on suicidality”* the following is fulfilled:

1) This material is the authors' own original work, which has not been previously published elsewhere.
2) The paper is not currently being considered for publication elsewhere.
3) The paper reflects the authors' own research and analysis in a truthful and complete manner.
4) The results are appropriately placed in the context of prior and existing research.
5) All sources used are properly disclosed (correct citation). Literally copying of text must be indicated as such by using quotation marks and giving proper reference.
6) All authors have been personally and actively involved in substantial work leading to the paper, and will take public responsibility for its content.

## Grant numbers and funding information

This study was financed in part by the Coordenação de Aperfeiçoamento de Pessoal de Nível Superior – Brasil (CAPES) – Finance Code 001. Additional grant support was provided by 10.13039/501100003593CNPq 303389/2016-8. The funders had no role in the study design or the decision to publish this article.

## Declaration of competing interest

None.

## Data Availability

No data was used for the research described in the article.
