# Supplementary material for: Meta-analysis of the effect of racial discrimination on suicidality
Source: SSM Popul Health. 2022 Nov 3;20:101283. doi: 10.1016/j.ssmph.2022.101283 (PMC9646655; doi:10.1016/j.ssmph.2022.101283)
Supplement: Multimedia component 1 [file mmc1.docx]

Supplementary material

PART 1.

Terms used for the systematic search

(Suicide OR Depression AND “Racial discriminat*” OR “Ethnic discriminat*” OR Racism OR “Discriminat* based on race” OR “Discriminat* based on ethnic*” OR “Race-based discriminat*” OR “Ethnic-based discriminat*” OR “Perceived discriminat*” OR “Race-related discriminat*” OR “Ethnic-related discrminat*” OR “Discriminat* related to race” OR “Discriminat* related to ethnici*”)
