# Supplementary material for: Meta-analysis of the effect of racial discrimination on suicidality
Source: SSM Popul Health. 2022 Nov 3;20:101283. doi: 10.1016/j.ssmph.2022.101283 (PMC9646655; doi:10.1016/j.ssmph.2022.101283)
Supplement: Multimedia component 2 [file mmc2.docx]

Supplementary material. PART 2

Table 1S – Complete data extraction of the 52 articles investigating the association between RD and suicidality

| **Authors** | **Sex/gender (% of females)** | **Age range** | **Mean age (SD)** | **Race/ethnicity** | **Sample size** | **Population** | **Sample recruitment** | **Study design** | **Suicidality Assessment** | **Timeframe of suicidality** | **Racial discrimination assessment** | **Timeframe of exposure to RD** | **Outcomes & findings** | **Observation** |
| --- | --- | --- | --- | --- | --- | --- | --- | --- | --- | --- | --- | --- | --- | --- |
| Diaz et al., 2001 | Male (0%) | NR | 31.2 (NR) | Latino | 912 | New York, NY; Los Angeles, LA; Miami, FL | Community sample | Cross-sectional | A four-level item on suicidality | Last 6 months | Ten binary questions on RD in different contexts | Lifetime | **1)** The following racial-related items were associated with SI: made fun of as a child (p =.0005), violence as a child (p = .0000), police harassment (p = .0049), rejected for sex (p = .0031); **2)** The following racial-related items were not associated with SI: made fun of as an adult (p= .145), treated rudely or unfairly (p=.0976), job discrimination (p=.0186), difficulty finding lovers (NS), objectifies sexually (p= .0176) |  |
| Freedenthal et al., 2004 | Male & Female (56.9%) | 13-20 | 15.6 (1.6) | Native American | 311 | Southwestern U.S. | American Indian Multisector Help Inquiry (AIM-HI) | Cross-sectional | One binary question on SA | Lifetime | One binary question on RD | Lifetime | In bivariate analyses, no association between RD and SA for participants residing in urban spaces, but significant association for those residing in reservations (p=.004) |  |
| Yoder et al., 2006 | Male & Female (46%) | 9-16 | 12.12 (1.44) | Native American | 201 | Reservations in Midwestern U.S. | Three Villages Project | Cross-sectional | One binary question on SI | Currently | Ten 3-point Likert scale on RD | Lifetime | **1)** In the bivariate analysis, RD associated with SI (B = 3.07, beta = 0.20, p < .001); **2)** Model 2: RD associated with SI (B = 3.47, beta = 0.29, p < .001); **3)** Model 3: RD associated with SI (B = 2.67, beta=0.25, p < .001); **4)** Model 4: RD not associated with SI (B = 2.08, beta = 0.19, p = .10) |  |
| Santana et al., 2007 | Male & Female (68.9%) | 10-21 | NR | Black & non-Black Brazilians | 973 | Salvador, BA, Brazil | Community sample | Cross-sectional | Two binary questions on SI and suicide planning | Lifetime | Four dichotomous items on RD. RD was coded as a binary variable if participants responded yes to any question | Lifetime | RD associated with SI (PRcrude = 1.57 95%CI:1.05–2.35) and with suicide planning (PRcrude =1.87, 95%CI:1.07–3.00) |  |
| Walls et al., 2007 | Male & Female (50%) | 10-13. | 11.1 (NR) | Native American | 721 | Midwest U.S. & Canada | Native reservations | Cross-sectional | One dichotomized item on suicidality | Last 12 months | Ten-item mean scored scale | Lifetime | **1)** In bivariate analysis, RD associated with suicidality (.14, p < .01); **2)** In Logistic Regression Models, Model 2: RD associated with suicidality (B .97, Exp b 2.63, p<0.05); Model 3: RD not associated with suicidality (B .75, Exp b 2.12); Model 4: RD not associated with suicidality (B .82, Exp b 2.26); Model 5: RD not associated with suicidality (B .68, Exp b 1.98) |  |
| Hwang & Goto, 2008 | Male & Female (63.9%) | Young adults | NR | Asian American & Latino | 186 | Rocky Mountains region, U.S. | Community sample | Cross-sectional | Scale for Suicidal Ideation (SSI, modified) | Current | General Ethnic Discrimination Scale | Lifetime | **1)** In the bivariate analysis, RD not associated with SI for Latinos (r=.10) or for Asian Americans (r=0.18); **2)** In the regression analysis, higher RD was significantly associated with SI, accounting for 3% of the variance (Beta 0.17, t2 = 2.28, p< 05, R2 = .03) |  |
| Borges et al., 2011 | Male & Female (58.45%) | 14-18 | NR | Hispanic, Non-Hispanic/Black/African American, Non-Hispanic/other, Non-Hispanic/White | 962 | Boston, MA | Boston Youth Survey (BYS) | Cross-sectional | One binary question on SI | Last 12 months | One binary question on RD | Last 12 months | **1)** RD associated with SI for US-born individuals (OR:2.1, 95%CI 1.2–3.8); **2**) RD not associated with SI for foreign-born individuals (OR: 1.2, 95% CI: 0.6–2.6) |  |
| Castle et al., 2011 | Male & Female (NR) | 18-24 | NR | African-American | 234 | Northeastern U.S. | Community sample | Cross-sectional | National Comorbidity Survey (NCS) items | Lifetime | The Racism and Life Experiences Scale (RaLeS) | Lifetime | RD not associated with SI (OR: 1.00, 95% CI: 0.99, 1.02) or SA (OR: 0.99, 95%CI: 0.96, 1.02) at a statistically significant level | Effect size provided by author |
| Gomez et al., 2011 | Male & Female (68%) | 18-25 | 18.8 (1.3) | Asian, Latino, Black & White | 969 | Northeastern U.S. | College students | Cross-sectional | One binary question on SA. If yes, how many attempts? | Lifetime | Schedule of Racist Events | Last 12 months | **1)** MODEL 1: RD not associated with SA (OR: 1.52, 95%CI 0.94 - 2.46); **2)** MODEL 2: RD associated with SA (OR: 5.76, 95%CI 1.10 - 30.08, 0 <.05); **3)** When using Model 1 and separating by ethnicity, RD associated with increased SA among Latinos (OR: 3.53, 95% CI: 1.17–10.63, p < .05) and non-US Whites (OR: 10.44 , 95% CI: 1.30–83.99, p < .05), but not for Asians (OR: 0.85, 95% CI: 0.36–2.00), Whites (OR: 5.92, 95% CI: 0.67–52.20), and Blacks (OR: 0.64, 95% CI: 0.09–4.59). |  |
| Hightow-Weidman et al., 2011 | Male (0%) | NR | 20.4 (NR) | African-American, Latino, and multiracial | 348 | Bronx, NY; Chapel Hill, NC; Chicago, IL; Detroit, MI; Houston, TX; Los Angeles, CA; Oakland, CA; and Rochester, NY | Special Projects of National Significance (SPNS) Initiative | Cross-sectional | One binary question on SI and one binary question on SA | Lifetime | Two three-point Likert items on racial bullying | Lifetime | Racial bullying not associated with SI (p = 0.15) or with SA (p= 0.55) |  |
| Jamieson et al., 2011 | Male & Female (NR) | ≅18 | NR | Australian Aboriginal | 336 | Northern Territory, Australia | Aboriginal Birth Cohort (ABC) | Cross-sectional | Strong Souls (Three binary questions on SI) | Past few months | One question on RD categorized into two groups (not really and little bits/fair bits/lots) | Lifetime | **1)** In bivariate analysis, RD associated with suicide risk (0.78, 95%CI 0.49-1.07, p < 0.05); **2)** RD associated with suicide risk in multivariate analyses (B = 0.34, 95%CI 0.08-0.60) | Same sample as Priest et al., 2011 |
| Priest et al., 2011 | Male & Female (53%) | 16-20 | 18.27 (1.06) | Australian Aboriginal | 345 | Northern Territory, Australia | Aboriginal Birth Cohort (ABC) | Cross-sectional | Strong Souls (Three binary questions on SI) | Past few months | One question on RD categorized into two groups (little bits and fair bits/lots) | Lifetime | **1)** In the bivariate analysis, strong association between RD and suicide risk (OR: 2.39, [95% CI 1.45–3.96], p = 0.001); **2)** In the multivariable model, strong associations remained between RD and suicide risk (OR: 2.32 [95% CI, 1.25–4.00]; p = 0.01) |  |
| Chao et al., 2012 | Male & Female (63%) | 18-53 | 23.14 (5.78) | African-American | 1555 | Midwestern U.S. | College students. Online survey | Cross-sectional | A five-level Likert item on SI | Current | The Presenting Problems Checklist (one 5-point Likert item) | Current | Racial discrimination associated with SI for women (Kendall’s tau-b = .22; X2d = 43.82, p < .001) and for men (Kendall’s tau-b = .18; X2d = 18.28, p < .001) |  |
| Luke et al., 2013 | Male & Female (56.4%) | 12-26 | 19 (4) | Australian Aboriginal | 172 | Melbourne, Australia | VAHS Young People’s Project (YPP) | Cross-sectional | One binary question on SI and one binary question on SA | Two weeks for SI / Lifetime for SA | One binary question on RD | Lifetime | RD associated with SI (95%CI: 53.0 - 82.0, p=0.028) and with lifetime SA (95%CI: 60.5 - 87.1, p < .001) |  |
| Pan & Spittal, 2013 | Male & Female (51.40%) | 13-15 | NR | Chinese (ethnicity not specified) | 8182 | Chinese cities of Beijing, Hangzhou, Wuhan, Urumqi | Global School-Based Health Survey (GSHS) | Cross-sectional | One binary question on suicidal plan and one on and SI | Last 12 months | One binary question on racial bullying | Last 30 days | **1)** Racial bullying associated with SI (Crude OR: 2.26, 95%CI: 1.23–4.16, p < 0.01), but not suicidal planning (Crude OR: 1.92, 95%CI: 0.81–4.54); **2)** In the adjusted model, racial bullying associated with SI (AOR: 2.12, 95% CI: 1.15–3.93, p < 0.05) but not with suicidal planning (AOR: 1.81, 95% CI: 0.76–4.33) |  |
| Polanco-Roman & Miranda, 2013 | Male & Female (80%) | 18-25 | 18.6 (1.19) | Asian, Black, Latino, White | 143 | Northeastern U.S. | College students | Longitudinal | 1) Beck Scale for SI; 2) One binary question on SA | Last week (SI) / Lifetime (SA) | The Schedule of Racist Events | Lifetime | **1)** Baseline RD not associated with follow-up SI (.08); **2)** MODEL 1: RD at baseline not associated with SI at follow-up (b = -0.02, SE 0.02, Beta = -0.10); **3)** MODEL 2: RD at baseline predicted SI at follow-up (b= -0.05, SE 0.02, Beta -0.19, p= .04); **4)** Indirect relation between perceived discrimination and SI through hopelessness statistically significant at a low Z Sobel=1.94, p=.05 but not at average Z Sobel=0.95, p=.34 |  |
| Thoma & Huebner, 2013 | Male & Female (33%) | 14-19 | 17.45 (1.36) | African-American, African-American mixed | 276 | Indianapolis, IN, Oakland, CA, Boston, MA, Philadelphia, PA | Diverse Adolescents Sexual Health (DASH) study | Cross-sectional | One binary question on SI | Last 12 months | Schedule of Racist Events | Last 12 months | **1)** In the bivariate analysis, RD correlated with SI (0.12, p < .05); **2)** in Separate Multivariate Regression Model, RD associated with SI (B: 0.763; SE: 0.313 p < .05); **3**) In a combined model with antigay discrimination, RD no longer associated with SI (B: 0.561, SE 0.363) |  |
| Tobler et al., 2013 | Male & Female (NR) | 17-18 | 18.1 (0.64) | African American, Hispanic, and White | 2490 | Chicago, IL | Community sample (schools) | Cross-sectional | A five-level Likert item on SI | Last 12 months | A two-item composite variable reflecting the frequency and intensity of RD | Lifetime | **1)** Occasional not disturbing RD associated with SI: (OR: 1.79, 95% CI: 1.09 - 2.50, p <.05); **2)** Occasional somehow disturbing RD not associated with SI (OR: 1.32, 95% CI: 0.69 - 1.95); **3)** Often disturbing RD associated with SI (OR: 2.65, 95%CI: 1.63 3.67, p <.01) |  |
| Garnett et al., 2014 | Male & Female (58.45%) | Adolescents, young adults | NR | Non-Hispanic White, Non-Hispanic Black, Hispanic, Non-Hispanic Asian, and other | 965 | Boston, MA | Boston Youth Survey (BYS) | Cross-sectional | One binary question on SI | Last 12 months | One binary question on RD | Last 12 months | No association between RD and SI OR: 1.29, 95% CI: 0.77–2.16, p= 0.327 | Same sample as Borges et al., 2011 |
| Walker et al., 2014 | Male & Female (48.6%) | 18-59 | 36.46 (12.47) | African-American | 236 | One southeastern community in the U.S. | Community sample | Cross-sectional | The Adult Scale for Suicide Ideation (ASIQ) | Last month | Index of Race-Related Stress-Brief | Lifetime | **1)** RD associated with SI (r = .13, p < .01); **2)** Although the overall effect RD was significantly associated with SI (beta = .16, SE = 0.07, p < .001), when depressive symptoms were statistically controlled, this association (i.e., the direct effect) was no longer significant (b = .06, SE = 0.07, p = .44), suggesting a full mediation |  |
| Brockie et al., 2015 | Male & Female (51%) | 15-24 | 19.25 (2.9) | Native American | 285 for SI, 279 for SA | One reservation (unidentified) in the U.S. | Community sample | Cross-sectional | One binary question on SA | Lifetime | Ten four-point Likert items on RD | Lifetime | No association between RD and SA (OR: 1.71, CI: 0.52, 5.58) | Effect size provided by author |
| Chesin & Jeglic, 2015 | Male & Female (86%) | ≅20 | NR | African American, White & other | 118 | Northeastern U.S. | College students. Online survey | Cross-sectional | Beck Scale for Suicide Ideation | Last week | The General Ethnic Discrimination Scale | Last 12 months | No association between ethnic discrimination and SI (B: 0.02, SE: 0.14, p = 0.88) |  |
| O'Keefe et al., 2015 | Male & Female (61.4%) | 18-48 | 19.65 (NR) | African-American, Asian American, Hispanic, Native American | 405 | Midwest U.S. | Undergraduate students. Online survey | Cross-sectional | The Hopelessness Depressive Symptom Questionnaire–Suicidality Subscale | Last 2 weeks | Racial Microaggressions Scale | Lifetime | **1)** Positive association between RD and SI (.13, p <.05); **2)** In the mediating analysis, RD affects SI through depressive symptoms (.14, p <.0001) |  |
| Perez-Rodriguez et al., 2014 | Male & Female (49.1%) | 18-65+ | NR | Hispanic | 6359 | U.S. (several areas) | National Epidemiologic Survey of Alcohol and Related Conditions (NESARC) | Cross-sectional | Two binary questions on SI and SA | Lifetime | One binary question on RD | Lifetime | RD associated with SI (beta = 0.051; p = < 0.001) and SA (beta = 0.020; p < .003) |  |
| Gattis & Larson, 2016 & 2017 | Male & Female (52%) | 16-24 | 20.60 (2.06) | African-American | 89 | Milwaukee, WI | Enrollment through an agency that provides services for homeless youth | Cross-sectional | Three binary questions on suicidal planning, SI, and SA. Suicidality was coded as binary variable if any participants responded yes to any question | Last 12 months | Experiences of Discrimination (adapted) | Last 12 months | PAPER ONE: **1)** In the bivariate analysis, RD correlated with suicidality (.31; p < .01); **2)** In the multivariate analysis, no association between RD and suicidality in any model. Model 1: OR 1.69 (SE .46), Model 2: OR 2.12 (SE .66), Model 3: OR 2.16 (SE .69), Model 4: OR 1.82 (SE .52), Model 5: OR 1.49 (SE .68)  PAPER TWO: No association between RD and SI in any model. Model 2: OR 1.29 (SE 0.32) [95% CI: 0.80, 1.09]; Model 3: OR 1.34 (SE 0.37) [95% CI: 0.77, 2.30]; Model 4: 1.22 (SE 0.36) [95% CI: 0.69, 2.16]; Model 6: OR 1.03 (SE 0.38) [95% CI 0.50, 2.13]; Model 7: OR 0.96 (SE 0.40) [95%CI 0.43, 2.16] | Two papers using the same sample |
| Sutter & Perrin, 2016 | Male & Female (53%) | NR | 29.5 (9.93) | African American, Latino, Asian, Native American & mixed race | 200 | U.S. (several areas) | Online survey | Cross-sectional | Suicidal Behavior Questionnaire | Last week | The Daily Life Experiences Scale (DLE) | Last 12 months | No direct (.14) or indirect effect (p= .095) of RD on SI |  |
| Wilson et al., 2016 | Trans*female | 16-24 | NR | African-American, Latino, White, Asian, mixed race | 216 | San Francisco Bay Area, CA | SHINE study | Cross-sectional | Five-point Likert item on suicidal thoughts | Last week | Experiences of Discrimination | Lifetime | Those with higher exposure to racial discrimination had significantly higher odds for stress related to thoughts of suicide than those with lower exposure (AOR 4.3, 95% CI 1.5–13.3) |  |
| Hollingsworth et al., 2017 | Male & Female (56.3%) | 18-27 | 19.45 (NR) | African-American | 135 | Midwest, U.S. | College students | Cross-sectional | The Hopelessness Depression Symptom Questionnaire- Suicidality Subscale | Last 2 weeks | Racial Microaggressions Scale | Lifetime | **1)** None of the six dimensions of Racial microaggression had an direct effect on SI (.10 / .06 / .08 / .05 / .00 / .02) ; **2)** Mediation analyses indicated that the racial microaggression dimensions of invisibility (B=.0038, SE= .0016, 95%CI: .0014 - .0085, Effect size: .0038), low-achievement/undesirable culture (B= .0021, SE= .0013, 95%CI: .0002 - .0051, Effect size= .0868), and environmental invalidations had an indirect effect on SI through perceived burdensomeness (B=.0021, SE= .0013, 95%CI= .0002 - .0051, Effect size: .0868); **3)** The RMs total score had an indirect effect on SI through perceived burdensomeness (point estimate = .0049, SE = .0047, 95% BC = .0010 to .0124), but not thwarted belonginess (point estimate = .0008, SE .0007, 95% BC .0003 to .0038) | Same sample as O’Keefe et al., 2015 |
| Li et al., 2018 | Male & Female (57.7%) | 60+ | 72.3 (8.3) | American Chinese | 3123 | Chicago, IL | Population-based Study of Chinese Elderly in Chicago (PINE) | Cross-sectional | **1)** Patient Health Questionnaire (item 9); **2)** Geriatric Mental State Examination-Version A | Last month | Experiences of Discrimination | Lifetime | **1)** In the bivariate analysis, RD correlated with SI (Pearson χ2 = 18.3, df = 1, p < .001); **2)** MODEL 1 (without adjustments): RD associated with SI (OR = 2.19, 95% CI = 1.48 – 3.24, Wald χ2 = 15.4, df = 1, p < .001); **3)** MODEL 2: RD associated with SI (OR=1.95, 95%CI: 1.22-3.09, p=.005); **4)** MODEL 3: RD associated with SI (OR:2.08, 95%CI: 1.33-3.26, p=.001); **5)** MODEL 4: RD associated with SI (OR: 2.34, 95%CI: 1.48- 3.71, p <.001); **6)** MODEL 5 (in the fully adjusted model): RD associated with SI (OR = 1.9, 95% CI = 1.18-3.08, y-standardized coefficient = .4, Wald χ2 = 6.9, df = 1, p = .01) |  |
| Arshanapally et al., 2018 | Male & Female (50%) | 13-32 | 17.9 (NR) | African-American | 796 | Missouri, MS | Missouri Family Study (MOFAM) | Cross-sectional | Three binary questions on suicidal plans, SI, and SA. Suicidality was coded as a binary variable if participants responded yes to any question | Lifetime | Experiences of Discrimination | Lifetime | **1)** MODEL 1: RD associated with suicidality (OR = 2.24; 95% CI: 1.47–3.41); **2)** Model 2: RD associated with suicidality when accounting for risk factors (OR = 1.76, 95% CI = 1.10–2.76). Risk factors are MDD, sexual abuse, and physical abuse; **3)** MODEL 3: when separating by sex, RD not associated with suicidality for men (OR: 1.52, 95% CI: 0.78–2.94) or for women (OR: 1.71, 95% CI: 0.93–3.14) | Effect size provided by author |
| Cardoso et al., 2018 | Male & Female (56.2%) | NR | 14.44 (2.23) | Latino | 534 | North Carolina | Community sample (schools) | Cross-sectional | Two binary questions on SI | Last 12 months | One binary question on racial bullying | Last 12 months | Racial bullying associated with SI via depression (b = 0.230, SE 0.055, p < .05) |  |
| Hong, 2019 | Male & Female (56.5%) | Over 19 | 42.6 (10.3) | Korean Chinese | 292 | Seoul and nearby areas, South Korea | Community sample | Cross-sectional | One binary question on SI | Last 12 months | One binary question on RD | Lifetime | No difference in the prevalence of SI between those with and without RD (11.5 vs. 5.7%, Chi square statistics 3.128, p = 0.077) |  |
| Hong et al., 2018 | Male & Female (61.2%) | 18-25 | 20.47 (1.83) | African-American, Asian American, Hispanic | 289 | Southwestern U.S. | Undergraduate students | Cross-sectional | Adult Scale for Suicide Ideation | Lifetime | Everyday Discrimination Scale | Lifetime | **1)** RD associated with SI in the bivariate analysis (r=.24, p <.01); **2)** Purpose in life was a significant moderator for the association between RD and SI (beta = -.11, p = .025; 95% CI -20, -.01); **3)** Ethnic identity was not a moderator for the association between RD and SI (beta = -.08, p =.13; 95% CI -.19, .03) |  |
| Kwon & Han, 2018 | Male & Female (49%) | NR | 37.35 (14.64) | Latino | 1916 | U.S. (several areas) | National Latino and Asian American Survey (NLAAS) | Cross-sectional | One binary question on SI | Lifetime | Three questions on a 4-point Likert scale | Lifetime | **1)** MODEL 2: increased odds of SI among those who reported RD (OR: 1.589; CI 1.237–2.042; p < 0.001); **2)** MODEL 3: RD remained associated with SI (OR:1.314, CI 1.005–1.716, p <0.05); **3)** MODEL 4: RD not associated with SI (OR: 1.117, CI 0.846 - 1.474); **4)** In the mediation analysis, RD not directly associated with SI (Estimates: 0.11, Robust SE 0.14), but SI was mediated by depressive disorder (Estimates: 0.05, Robust SE: 0.02, mediation 24.68%, p < .05) | Effect size provided by author |
| Williams et al., 2018 | Male & Female (54.4%) | 12-19 | NR | Maori | 1623 | New Zealand | Youth'12 | Cross-sectional | One binary question on SA | Last 12 months | Three binary questions on ethnic discrimination | Lifetime | **1)** In univariate analysis, RD associated with SA (OR: 2.23, 95%CI: 1.59–3.14, p<.05); **2)** In multivariate model, those experiencing ethnic discrimination were more likely to have attempted suicide (aOR: 2.47, 95% CI 1.71–3.58, p <.05) |  |
| Madubata et al., 2022 | Male & Female (49%) | 14-17 | 15.1 (0.53) | African-American & Latinx | 157 | Southeast U.S. | Community sample | Longitudinal | A five-point 10-item scale that assesses suicidal thoughts | Last 3 months | The Way Discrimination Scale | Last 6 months | **1)** In bivariate correlations, subtle RD associated with baseline SI (.29, p < .001 ) and marginally associated with follow-up SI (.22, p < .05); **2)** In bivariate correlations, overt RD not associated with baseline SI (.14) and follow-up SI (.7); **3)** When separating by race, subtle RD associated with baseline SI (.30, p <.01) and follow-up SI (.39, p < .01) for African American, but not for Latinx (.22 & -.10); **4)** Overt RD not associated with baseline SI (.21) and follow-up SI (.22) for African Americans, but also not for Latinx (.08 & -.10); **5)** In the path analyses, subtle RD predicts follow-up SI (0.33 (.11), p <.01), but not overt RD (-0.06 (.12)) |  |
| Polanco-Roman et al., 2019 | Male & Female (72%) | 18-29 | 19.88 (2.25) | African-American, Hispanic/Latino, White, Asian & other | 1344 | Northeastern U.S. | College students | Cross-sectional | The Suicidal Behaviors Questionnaire-Revised | Last 12 months | General Ethnic Discrimination Scale | Last 12 months | **1)** Positive correlation between RD and SI (r = 0.09); **2)** There was a significant indirect effect of racial/ethnic discrimination on SI through traumatic stress to depressive symptoms in men, b = 0.002, 95%CI = 0.001–0.004, and women, b = 0.005, 95%CI = 0.003–0.006; **3)** Indirect effect of RD on SI through depressive symptoms in men, b = 0.003, 95%CI = 0.0004–0.006, but not in women, b = 0.001, 95%CI = −0.001 to 0.004 |  |
| Choi et al., 2020 | Male & Female (56.35%) | 11-19 | 15 (1.91) | Asian American (Korean & Filipino) | 786 (baseline) | Chicago area, IL | Midwest Longitudinal Study of Asian American Families (MLSAAF) | Longitudinal | One binary question on SI | Last 12 months | Five items on a 5-Likert scale from the MLSAAF project, ranging from 1 (almost never) to 5 (very often) | Lifetime | **1)** In the model adjusted for minority status, RD associated with SI (OR = 2.61, p < .001); **2)** In the saturated models, RD associated with SI (OR = 1.85, p < .001) | Effect size provided by author |
| Edwards et al., 2020 | Male & Female (54.9%) | 12-18 | 13.88 (1.30) | American Indian & Alaska Native | 400 | Great Plains, U.S. | Community sample | Cross-sectional | One binary question on SI | Last 6 months | One binary question on racial bullying | Lifetime | RD associated with SI (r = .18 p < .05) |  |
| Layland et al., 2020 | Male & Female (50.8%) | 18-68 | 39.1 (12.3) | Non-Hispanic Black and Hispanic | 817 | U.S. (several areas) | National Epidemiologic Survey on Alcohol and Related Conditions (NESARC) | Cross-sectional | One binary question on SA | Last 5 years | Experiences of Discrimination | Lifetime | RD not associated with SA. Age-stratified investigation: 18-25 years of age: 1.81 (0.60–5.49); 26-35 years of age: 1.72 (0.38–7.85); 36-60 years of age: 0.81 (0.20–3.27) |  |
| Taliaferro et al., 2020 | Male & Female (56%) | 18-26 | NR | Asian, European, Hispanic/Latino, African, Middle Eastern, and other | 435 | Midwestern & Southeastern, U.S. | Foreign college students | Cross-sectional | Two five-level Likert items on SI | Last 12 months | Discrimination Stress Scale | Last 12 months | **1)** Greater SI associated with higher ethnic discrimination (r=.24); **2)** in a multivariate regression analysis, no association found (no data shown) |  |
| Argabright et al., 2021 | Male & Female (47.7%) | 9-10 | 10.9 (0.64) | White, Black, Asian, American Indian, Native Hawaiian & other | 11235 | U.S. (several areas) | The ABCD Study | Cross-sectional | Single binary measure termed “suicidality" | Last 12 months | Seven five-level Likert items on RD | Last 12 months | **1)** Basic model: RD associated with suicidality (OR = 3.5, 95% CI = 2.95-4.16, p <.001); **2)** Main model: RD associated with suicidality (OR = 2.6, 95%, CI = 2.1-3.21, p <.001); **3)** Exposome model: RD associated with suicidality (OR =1.8, 95% CI = 1.43-2.26, p <.001); **4)** Psychopathology model: RD associated with suicidality (OR = 1.55, 95% CI = 1.22-1.96, p <.001); **5)** Full model: RD not associated with suicidality (OR = 1.24 0.97-1.59, p = .091) |  |
| Galán et al., 2021 | Male & Female (53.7%) | NR | 15.7 (1.3) | White, Black, or African American, Asian, American Indian, or Alaska Native, Native Hawaiian or Other Pacific Islander, and other | 3650 | Pittsburgh, PA | Public schools | Cross-sectional | One binary question on SI | Lifetime | One binary question on racial bullying | Last 12 months | Racial bullying associated with increased SI (aOR: 1.65; 95% CI, 1.19-2.29) | Effect size provided by author |
| Goodwill et al., 2021 | Male (0%) | 18-93 | 43.53 (16.18) | African-American | 1271 | U.S. (several areas) | National Survey of American Life (NSAL) | Cross-sectional | One binary question on SI | Lifetime | Everyday Discrimination Scale | Lifetime | **1)** RD correlated with SI .091 p < .001; **2)** In the path model, RD associated with SI (beta = .132, p < .048); 3) indirect effect from Race-Based Everyday Discrimination to SI via depressive symptoms was statistically significant (beta = .027, p = .033; 95% CI, .002–.052). |  |
| Lacey et al., 2021 | Female (100%) | NR | 42.5 (NR) | African American & Caribbean Black | 3277 | U.S. (several areas) | National Survey of American Life (NSAL) | Cross-sectional | One binary question on SI | Lifetime | Major Experiences of Discrimination. | Lifetime | Model 2: RD not associated with SI (OR: 1.144 [CI 0.996–1.314]); Model 3: RD not associated with SI (OR: 1.092 [CI 0.940–1.269]) | Effect size provided by author |
| Mallory & Russel, 2021 | Male & Female (49%) | 15-24 | 19 (1.79) | Black, Latino, Multi-racial, Native American, Asian, Hawaiian, and Pacific Islander | 585 | One city in the Northeast and one in the Southwest, U.S. | Community sample | Longitudinal | Positive and Negative Suicide Inventory | Last 2 weeks | Brief Racism and Life Experience and Stress Scale (RaLS-B) | Last 12 months | **1)** Baseline RD positively associated with baseline SI (b = 0.26, SE 0.07, p < 0.001); **2)** Baseline RD associated with SI at wave 2 b = 0.15, p 0.01, but not at wave 3 (b=0.09 and at wave 4 (b= 0.09); **3)** In the sensitive analysis (alternative model), baseline RD had a main effect on baseline SI (beta=0.14, SE 0.05, p < 0.01); **4)** Baseline RD had no main effect on SI overtime (beta=0.09, SE 0.05 / beta = 0.09, SE 0.07 / beta = 0.02, SE 0.07) |  |
| Oh & Nicholson, 2021 | Male & Female (NR) | 18+ | NR | African Americans & Caribbean Black Americans | 5191 | U.S. (several areas) | National Survey of American Life (NSAL) | Cross-sectional | Two binary questions on SI and SA | Lifetime | Two five-point items on RD (colorism) | Lifetime | **1)** In bivariate models, intra-group RD associated with SI (OR: 1.16, 95% CI: 1.06-1.27, p <0.05), but not inter-group RD (OR:1.05, 95%CI: 0.96-1.14); **2)** In bivariate models, intra-group RD associated with SA (OR: 1.16, 95% CI: 1.01 - 1.3, p<0.05); 2), but not inter-group RD (OR: 1.03, 95%CI: 0.88-1.20); **3)** In adjusted models, intra-group RD significantly associated with greater odds of lifetime SI [aOR: 1.21; 95% CI: 1.08-1.36 p<0.05 ] and SA [aOR: 1.22; 95% CI: 1.04-1.44, p=.00]; **4)** In adjusted models, inter-group RD not associated with SI [aOR: 0.97, 95% CI: 0.87- 1.08] and with SA [aOR: 0.95, 95% CI: 0.78 - 1.16] |  |
| Okoye & Saewyc, 2021 | Male & Female (48%) | NR | 15.1 (NR) | African Canadians | 535 for SI, 533 for SA | British Columbia, Canada | British Columbia Adolescent Health Surveys (BCAHS) | Cross-sectional | Two binary questions on suicidal thoughts and SA | Last 12 months | One binary question on RD | Last 12 months | **1)** RD associated with increased risk of SI (AOR: 1.97, 95% CI = 1.32 - 2.94, p < .01) and SA (AOR: 2.05, 95% CI = 1.10 - 3.83, p < .05); **2)** Stratified by gender, RD associated with SI for girls (AOR: 2.01, 95% CI = 0.83 - 2.83, p < .0.01), but not for boys (AOR: 1.53, 95% CI = 1.20 - 3.34); **3)** RD associated with SA for boys (AOR: 2.94, 95% CI = 1.09 - 7.91, p < .05), but not for girls (AOR: 1.61, 95% CI = 0.72 - 3.60); **4)** For the Canadian-Born, RD not associated with SI (AOR: 1.54, 95% CI = 0.98 - 2.42) or with SA (AOR: 1.64, 95% CI .80 - 3.36); **5)** For immigrants, RD associated with SI (AOR: 4.66, 95% CI = 2.26 - 9.58) and with SA (OR: 3.94, 95% CI = 1.15 - 13.55) | Effect size provided by author |
| Polanco-Roman et al, 2021 | Male & Female (61%) | 18-29 | 19.84 (2.22) | Asian, Hispanic, Black & other | 747 | Northeast U.S. | College students | Cross-sectional | The Self-Injurious Thoughts and Behaviors Interview | Last 12 months (SI) & lifetime (SA) | Experiences of Discrimination | Lifetime | **1)** In the bivariate analyses, RD associated with SI (r=.08, p <.05) but not with SA (r=.04); **2)** In the fully adjusted model, no association of RD with SI (b (SE)= 0.01 (0.02), 95%CI [ 0.05, 0.03], p= .71) or with SA (b (SE) = 0.003 (0.004), 95% [ 0.01, 0.01] p = .57) |  |
| Wang et al., 2021 | Male & Female (76%) | Adults | 44.47 (15.65) | Non-Hispanic Black, non-Hispanic American Indian/Alaska Native, non-Hispanic Asian/Native Hawaiian/Other Pacific Islander, and Hispanic | 960 | U.S. (several areas) | National Epidemiologic Survey on Alcohol and Related Conditions (NESARC) | Longitudinal | Two binary questions on SI and SA | Lifetime | Six questions on RD rated in a 5-point Likert scale (three on institution discrimination and three on interpersonal discrimination) | Lifetime | LOGISTIC REGRESSION MODELS: **1)** Institutional RD at wave 1 predicted onset of SI at wave 2 (OR = 1.67, 95% CI:1.05–2.66, p < 0.05); **2)** SI related to institutional RD at wave 1 predicted SA at wave 2 (OR = 2.67, 95% CI:1.30–5.48, p < 0.05); **3)** interpersonal RD at wave 1 predicted onset of SI and SA at wave 2 (OR = 2.34, 95% CI:1.17–4.66, p < 0.05); **4)** Institutional RD at wave 1 did not predict onset of SI and SA at wave 2 (OR = 0.09, 95% CI: 0.01-1.76); **5)** Interpersonal RD at wave 1 did not predict onset of SI at wave 2 (OR: 0.98, 95% CI: .69-1.39); 6) SI related to institutional RD at wave 1 did not predict SA at wave 2 (0.69, 95% CI: 0.23-2.10) |  |
| Zimmerman & Miller-Smith, 2022 | Male & Female (50.31%) | 9-12 | 10.61 (1.54) | Hispanic, African-American, White, Asian, Pacific Islander, Native American, mixed-race | 1147 | Chicago, IL | Project on Human Development in Chicago Neighborhoods (PHDCN) | Longitudinal | One dichotomized item on suicidality | Last 12 months | Eight dichotomous items for each construct: Experienced RD, Anticipated RD, Vicarious RD | Lifetime | **1)** Experienced RD associated with suicidality (OR = 1.30; 95 % CI = 1.13, 1.50 p < .001); **2)** Anticipated RD associated with suicidality (OR = 1.18; 95 % CI = 1.05, 1.33 p < .01); **3)** Vicarious RD associated with suicidality (OR = 1.18; 95 % CI = 1.07, 1.30 p < .001); **4)** In the combined model, Experienced RD associated with suicidality (OR = 1.24; 95 % CI = 1.04, 1.48 p < .05), as Vicarious RD (OR = 1.14; 95 % CI = 1.03, 1.26 p < .05), but not Anticipated RD (OR = 1.08; 95 % CI = 0.92, 1.26); **5)** Women are at increased risk of showing suicidality (OR = 2.05; 95 % CI = 1.34, 3.13) | Effect size provided by author |
